# Flexible TiO_2_/ZrO_2_/AuCNAs Surface-Enhanced Raman Scattering Substrates for the Detection of Asomate in Apple Peel

**DOI:** 10.3390/foods14122062

**Published:** 2025-06-11

**Authors:** Lina Zhao, Zhengdong Sun, Ye Shen, Zhiyang Chen, Yang Zhang, Jiyong Shi, Haroon Elrasheid Tahir, Xuechao Xu, Meng Zhang, Xiaobo Zou, Kaiyi Zheng

**Affiliations:** 1 School of Food and Biological Engineering, Jiangsu University, Zhenjiang 212013, China; 2222218049@stmail.ujs.edu.cn (L.Z.); shenye@stmail.ujs.edu.cn (Y.S.); 15370988685@163.com (Z.C.); yangzhang1@ujs.edu.cn (Y.Z.); shi_jiyong@ujs.edu.cn (J.S.); haroona28@yahoo.com (H.E.T.); 2 Department of Physics, East China University of Science and Technology, Shanghai 200237, China; y30231297@mail.ecust.edu.cn (Z.S.); mzhang@ecust.edu.cn (M.Z.); 3 School of Food Science and Engineering, Yangzhou University, Yangzhou 225127, China; xuechaoxu@yzu.edu.cn

**Keywords:** SERS, asomate, flexible substrate, electrospinning, non-destructive detection

## Abstract

(1) Background: Asomate, as a dithiocarbamate compound, is moderately toxic to the human body; thus, it is necessary to develop a rapid and efficient method for detection. To meet this need, this study introduced a rapid, non-destructive, and efficient method for detecting asomate residues on the surface of apples based on surface-enhanced Raman spectroscopy (SERS) combined with flexible substrates. (2) Methods: Concave Au nanorods (AuCNAs) were synthesized in advance. Then, the AuCNAs were loaded on an electrostatically spun film to generate a flexible TiO_2_/ZrO_2_/AuCNAs substrate for detection. (3) Results: The flexible substrate exhibited strong SERS activity, with an enhancement factor (EF) up to 9.40 × 10^7^ for 4-MBA. Meanwhile, the finite-difference time-domain (FDTD) simulation showed that the localized surface plasmon resonance (LSPR) effects related to the enhancement of the SERS signal are mainly generated from the ‘hot spots’ in AuCNAs. The density functional theory (DFT) simulation detailedly revealed that the SERS peaks could be generated by the interaction among asomate molecules, disassociated Au atoms, and Au facets. Moreover, the asomate in apple peel was analyzed with the limit of detection (LOD) as low as below 10 nM, allowing for the rapid detection of asomate directly on apple peels. (4) Conclusions: The flexible TiO_2_/ZrO_2_/AuCNAs film can be used for the in situ detection of asomate in apple peel at low concentrations. Moreover, the simulation methods, including FDTD and DFT, explained the mechanism of SERS from the flexible substrates.

## 1. Introduction

Apples are among the most widely cultivated and consumed fruits globally [1,2]. Various fungicides are used at different stages of apple growth to control infections and maintain quality. Dithiocarbamate compounds are commonly used to control fungi [3]. Asomate, a class of dithiocarbamate compounds, has strong antifungal properties and long persistence [4]. It has effectively controlled various plant diseases and was widely used [5]. However, asomate is moderately toxic and can be decomposed to generate more toxic compounds in the human body to cause liver, kidney, and nerve illnesses [6,7]. Therefore, it is necessary to monitor the residues of asomate in apples.

At present, the primary detection methods for asomate are gas chromatography combined with mass spectrometry (GC-MS) [8] and high-performance liquid chromatography combined with mass spectrometry (HPLC-MS) [7,9]. Although those methods are quantitatively accurate, they are cumbersome to operate [10], and the corresponding equipment is costly and unsuitable for on-site detection. Therefore, there is a need to develop rapid and low-cost techniques for on-site detection. Surface-enhanced Raman spectroscopy (SERS) is an analytical technique that amplifies Raman signals through roughened metallic nanoparticles [11]. Compared with other methods, high specificity, rich molecular information, good sensitivity, and ease of operation are the unique advantages of SERS [12]. This technique has found extensive applications in agricultural [13,14,15], environmental [16,17,18], and biomedical fields [19,20,21]. Among the different SERS substrates, traditional rigid and sol–gel substrates, although widely used, cannot meet the non-destructive testing of samples with rough and uneven detection surfaces [22], such as the apple surface. Thus, to meet the requirements of detection scenarios, flexible materials such as paper, polymer films, adhesive tapes, and biological tissues have attracted considerable attention in the construction of flexible SERS substrates [23]. These substrates are highly portable and can perfectly adhere to curved surfaces [24]. Meanwhile, flexible SERS substrates have numerous advantages over traditional substrates in terms of sample handling and detection operation. They can be rapidly sampled and even detected in situ on the surface by simple lamination.

Electrostatic spinning is a specialized fiber manufacturing technique that enables the fabrication of fibers with uniform film formation, high porosity, superior mechanical properties, and specific surface area [25], rendering them suitable for various applications [26,27,28]. Regarding SERS detection, Yi et al. [29] fabricated a polyurethane film through electrostatic spinning. Subsequently, they coated the same with ultrathin silver nanolayers with vacuum evaporation, generating a SERS substrate for detecting aflatoxins. Wei et al. [30] deposited AuNS@Ag onto electrostatically spun thin films, fabricating a homogeneous and flexible SERS substrate to detect carbendazim in apple peel.

In recent years, noble metal and semiconductor-natured substance composite materials such as SERS substrates have showcased remarkable advantages in analytical and detection applications, including enhanced sensitivity, stability, and recyclability. Meng et al. [31] demonstrated that TiO_2_ nanofilms with a nanoparticle structure can be in situ grown on aluminum sheets using a simple sol–hydrothermal method. These films exhibit excellent stability and maintain a stable SERS signal at room temperature for over 20 weeks. Although TiO_2_ has strong optical properties, it still has a small specific surface area and poor adsorption capacity [31,32], while the energy band gap of ZrO_2_ is too large, making charge separation difficult [33,34]. The synthesized TiO_2_/ZrO_2_ composites, upon doping, exhibit a high specific surface area, strong adsorption capacity, and excellent photocatalytic properties. Thus, nanofibers synthesized from composite semiconductor materials have garnered significant research interest [35,36,37]. Concurrently, noble metal nanoparticles (NPs), such as Ag, Au, and Cu, integrated with TiO_2_/ZrO_2_ composites, exhibit a strong localized surface plasmon resonance (LSPR) effect under laser irradiation, resulting in intense SERS signals [38]. Furthermore, the Au nanoparticles (AuNPs), TiO_2_, and ZrO_2_ all demonstrate excellent biocompatibility. Au NPs are well-suited for constructing drug delivery systems [39], as they are readily excreted renally and do not elicit inflammatory responses in human tissues [40,41]. TiO_2_ and ZrO_2_ are also recognized as safe for human exposure. First, TiO_2_ is a permitted food additive widely incorporated into food matrices [42]. Additionally, both TiO_2_ and ZrO_2_ find application in food packaging [43] and serve as biomedical materials within dentistry [44], orthopedics [45], nursing [46], and pharmaceutics [47,48]. Consequently, composite films incorporating noble metals and semiconductor materials, such as TiO_2_/ZrO_2_/AuNPs, represent specialized candidates for flexible SERS substrates.

**Figure 1:**
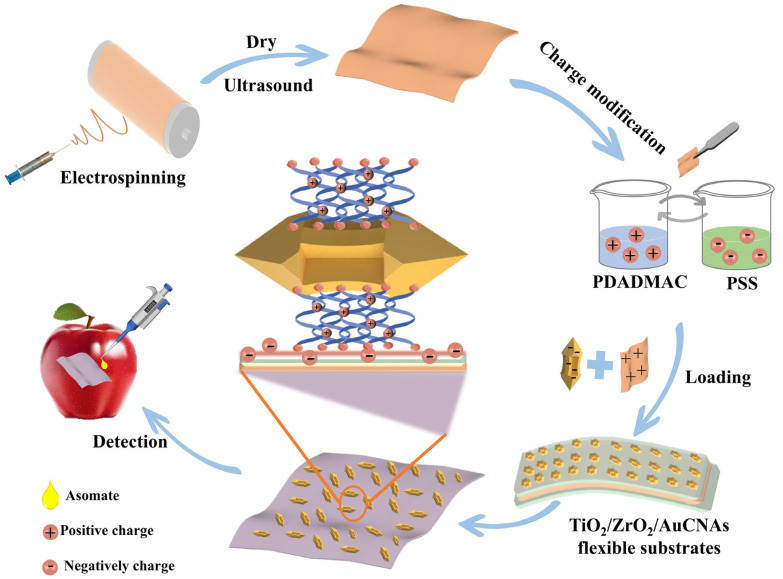
Schematic illustration of the synthesis procedure for TiO_2_/ZrO_2_/AuCNAs films and their application in detecting fungicides on apple surfaces using SERS.

This study illustrates the preparation of TiO_2_/ZrO_2_ films through electrospinning in Figure 1. Concave Au nanorods (AuCNAs) were then loaded onto the films through an electrostatic adsorption assembly strategy to form a uniformly flexible SERS substrate. The newly developed flexible SERS substrate demonstrated remarkable Raman enhancement capabilities and was utilized to detect asomate on the surface of an apple.

## 2. Materials and Methods

### 2.1. Reagents and Apparatus

In this study, sodium borohydride (NaBH_4_), silver nitrate (AgNO_3_), hydrogen tetrachloroaurate (III) trihydrate (HauCl_4_·3H_2_O), and sodium oleate (NaOL) were sourced from Merck Reagents (Rahway, NJ, USA). Additional reagents, including hexadecyl trimethyl ammonium bromide (CTAB), hexadecyl trimethyl ammonium chloride (CTAC), hydrochloric acid (HCl, 37 wt%), L-ascorbic acid (AA), acetone, TiO_2_, ZrO_2_, poly dimethyl diallyl ammonium chloride (PDADMAC, 15,000 g/mol, 20 wt%), polystyrene sulfonic acid (PSS, 70,000 g/mol, 30 wt%), polyvinyl pyrrolidone (PVP), and N, N-dimethylacetamide (DMAC) were obtained from China Pharmaceutical Chemical Reagents Co., Ltd. (Shanghai, China). Polyvinylidene difluoride (PVDF), asomate, carbendazim, chlorpyrifos, thiophanate-methyl, acetamiprid, imidacloprid, and captan were obtained from Genye Biologicals (Guangzhou, China) and Aladdin (Shanghai, China), respectively. Apples were acquired from a retail store in Zhenjiang, China.

The instruments used for materials characterization include the ultraviolet–visible (UV-vis) absorption spectrometer (T6, Beijing Puxi General Instrument Co., Ltd., Beijing, China), transmission electron microscopy (TEM, JEM-2100F, JEOL Ltd., Tokyo, Japan), thermal field emission scanning electron microscopy (SEM, JSM-7001F, JEOL Ltd.), X-ray diffraction (XRD, SmartLab, Rigaku Ltd., Tokyo, Japan), and zeta potential and nanoparticle analyzer (Nano-ZS90, Malvern Instruments Ltd., Malvern, UK). The Raman spectra in the range of 300~2000 cm^−1^ were collected using a Raman spectrometer (XploRA PLAS, HORIBA, Palaiseau, France) at an integration time of 5 s under 785 nm laser excitation and 100 mW power.

### 2.2. Synthesis of AuCNAs

Au nanorods (AuNRs) were synthesized using the seed growth method [49]. In detail, the ice-cold NaBH_4_ (0.6 mL,10 mM) was added to the beaker containing CTAB (10 mL, 0.1 M) and HAuCl_4_ (0.25 mL, 10 mM) under magnetic stirring. The resulting solution turned brown, after which it was allowed to stand at 27 °C for 2 h to generate the Au seeds. Subsequently, the growth solution was prepared by ultrasonically dissolving CTAB (1.351 g) and NaOL (0.305 g) in deionized water (100 mL). After that, the AgNO_3_ solution (1.92 mL, 10 mM) was added, and the static reaction time was kept at 15 min. Next, 5 mL of HAuCl_4_ (10 mM) was added slowly. After two hours, the HCl solution (600 μL, 37 wt%) was added. After adding AA (600 μL, 0.02 M) and stirring vigorously for 30 s, the solution was combined with 40 μL of the prepared Au seed. The resulting solution was stored overnight at 27 °C in a water bath. The AuNRs were collected by centrifugation at 6500 rpm for 15 min and repeated twice.

AuCNAs were prepared using the overgrowth method [50,51]. Briefly, 10 mM HAuCl_4_ solution (0.3 mL) was first added to a CTAC solution (0.1 M, 10 mL) under stirring. After the sequential addition of AgNO_3_ (10 mM, 0.25 mL), HCl (1 M, 0.2 mL), and AA solution (0.1 M, 0.15 mL), the solution was vigorously stirred for 30 s. Finally, 0.5 mL of the 20-fold concentrated AuNRs solution was injected. The resultant mixture was stirred for 10 s and left undisturbed for 4 h at 30 °C for the growth of AuCNAs. After two centrifugation cycles at 6500 rpm for 15 min, the product was stored in the refrigerator at 4 °C.

### 2.3. Electrospinning of Nanofiber Films with TiO_2_ and ZrO_2_

Flexible SERS substrates were prepared based on the previous literature, with modifications [26]. The 1.04 g of PVDF and 0.52 g of PVP powders were dissolved in 10 mL of DMAC/acetone (1:1/*v*:*v*) and then configured. Subsequently, TiO_2_ and ZrO_2_ were added to the solution in varying mass ratios (TiO_2_:ZrO_2_ = 0:6, 1:5, 2:4, 3:3, 4:2, 5:1, 6:0 wt%) and stirred magnetically for 12 h to prepare the electrospinning solution. The electrospinning process was performed with the supplied voltage of 20 kV and a working distance of 15 cm. The advance rate of syringes was 0.002 mm/s. The nanofiber film was collected on aluminum foil paper. To remove PVP and PVDF and create a porous structure, the nanofiber film was subjected to the following treatments: ultrasonic processing for 1.5 h, soaking in water at 60 °C for 24 h, and drying at 45 °C.

### 2.4. Assembly of Flexible SERS Substrate

Layer-by-layer assembly was carried out in the previous literature with modifications [52]. Firstly, the nanofiber film was alternately immersed in solutions containing PDADMAC-NaCl (10 g/L) and PSS-NaCl (20 mL), respectively, each for 30 min to transform the charge and repeat the layer-by-layer deposition. Ultimately, this process resulted in the aggregation of PDADMAC on the membrane surface, which had acquired a positive charge deposition.

The synthetic AuCNAs were immersed in a PSS-NaCl (3 g/L) solution for one hour to load negative charges on their surface. The negative charges not loaded onto the AuCNAs were removed by centrifugation (6500 rpm, 10 min), and the AuCNAs were redispersed in ultrapure water. Subsequently, the modified nanofiber film was immersed in AuCNAs colloid for 0, 1, 2, 4, 6, 8, 10, 12, and 14 h. Following the loading of AuCNAs, the nanofiber film was rinsed with ultrapure water for three minutes and subsequently dried at 45 °C. Finally, TiO_2_/ZrO_2_/AuCNA flexible SERS substrates were synthesized.

### 2.5. Detection of Standard Solutions

An asomate solution with a concentration of 1 mM in acetone was prepared to serve as a standard stock solution. A series of asomate solutions with different concentrations (50 nM~1 μM) were prepared by diluting the standard stock solution with acetone. For SERS measurements, 2.5 μL of asomate standard solution was added dropwise onto the flexible SERS substrates loading AuCNAs, and then Raman spectra were collected, with three measurements for each sample.

### 2.6. Detection of Actual Samples

The detection procedure was based on previous reports [53]. The apple samples were washed with ultrapure water. Subsequently, asomate solutions at different concentrations (100 nM~1 μM) were sprayed onto the apple epidermis. The solution was then allowed to dry at an ambient temperature. Subsequently, 10 μL of absolute ethanol was added dropwise onto the blot for dissolution. After that, the AuCNAs-assembled flexible SERS substrate was applied to the apple imprint for 60 s and then peeled off. Finally, a SERS analysis was carried out using the Raman spectrometer.

### 2.7. Data Analysis for Detection

Smooth and noise reduction were performed using LabSpec6 spectroscopy software. The limit of detection (LOD) of asomate is based on the method of Pu et al. [54]:

(1) $$LOD=\frac{3\;\mathrm{SD}}{k}$$

Here, SD in Equation (1) denotes the standard deviation of the SERS intensity of the blank samples, while k is used to represent the slope of the calibration curve.

The experimental enhancement factor (EF) is calculated according to Equation (2):

(2) EF=ISERSNSERSIRamanNRaman

In Equation (2), $I_{\mathrm{SERS}}$ and $I_{\mathrm{Raman}}$ are the intensities of the characteristic peaks of probe molecules in SERS and normal Raman spectra, while $N_{\mathrm{SERS}}$ and $N_{\mathrm{Raman}}$ denote the number of probe molecules contributing to SERS and normal Raman, respectively.

### 2.8. Theoretical Simulations

The three-dimensional (3D) finite-difference time-domain (FDTD) simulations were executed through Lumerical FDTD Solutions 2020 software to investigate the electromagnetic field distribution of nanoparticles in flexible substrates, including TiO_2_, ZrO_2_, and AuCNAs. These simulations provided information related to the electromagnetic field distribution, allowing for exploring the LSPR effects on the SERS substrates.

The density functional theory (DFT) calculations combined with the Cambridge Serial Total Energy Package (CASTEP) module were employed, based on the first-principles quantum mechanical program grounded in density functional theory, to optimize the geometry of asomate adsorbed on the Au (111) surface. This step was crucial for ensuring the precision of our subsequent analyses. The Perdew–Burke–Ernzerhof (PBE) functional, within the framework of the generalized gradient approximation (GGA), was chosen to model electron exchange and correlation interactions. The plane-wave cutoff energy was set at 500 eV. To address the van der Waals interactions between the adsorbate and the Au (111) surface, the Tkatchenko and Scheffler (TS) dispersion correction was applied. The energy convergence criterion was set to a stringent 1.0 × 10^−5^ eV/atom, with maximum force and stress thresholds defined at 0.05 eV/Å and 0.05 GPa, respectively. The maximum atomic displacement was constrained to 0.001 Å to maintain computational rigor. A 20 Å vacuum layer was introduced to isolate the system to mitigate the influence of interlayer coupling.

Employing the Gaussian 16 software suite, grounded in DFT for assessing Raman activity, the B3LYP functional approach, in conjunction with the 6-31G (d) basis set, was utilized for the small atoms, encompassing C, H, N, S, and As. For Au atoms, the LANL2DZ basis set was selected. Subsequent vibrational frequency analyses of all optimized structures were conducted by employing the B3LYP functional approach.

## 3. Results and Discussion

### 3.1. Characterization of AuCNA

**Figure 2:**
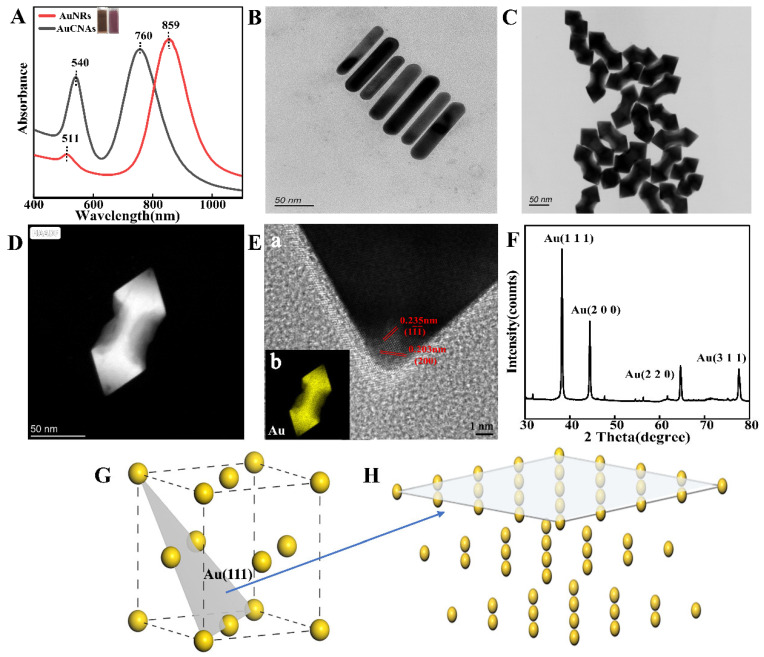
(**A**) UV-vis spectra of AuNRs and AuCNAs; (**B**) TEM images of AuNR; (**C**) TEM images of AuCNAs; (**D**) HAADF-STEM images of AuCNAs; (**E**) Au elemental distribution (b) and HRTEM (a) images of AuCNA; (**F**) XRD patterns of AuCNAs; (**G**) the draft of Au face-centered cube; (111) Miller plane; and (**H**) the draft of Au (111) surface.

The UV-Vis absorption spectra of the AuNRs, depicted in Figure 2A (black curve), exhibit characteristic transverse and longitudinal peaks at 511 nm and 859 nm, respectively. The dimensions of the AuNRs, determined using Nano Measurer 1.2.5 software, revealed an average length of 85 ± 4.26 nm and a diameter of 20.3 ± 1.92 nm, yielding a length-to-diameter ratio of 4.2:1. Notably, the UV-Vis spectra of the AuNRs demonstrate a blue shift in the longitudinal absorption band from 859 nm to 760 nm, while the transverse absorption peak experiences a red shift from 511 nm to 540 nm. This phenomenon can be attributed to a reduction in the aspect ratio of the AuNRs during the formation of a side depression and a double-conical tip structure. This structural transformation occurs under the influence of selective deposition growth induced by the surfactant of CTAC [55], which shows varied optical features and strong absorption [56]. As shown in the TEM image in Figure 2B, uniform AuNRs with individual nanoparticle lengths ranging from 80 to 95 nm are presented. As shown in Figure 2C, the morphology of AuCNAs presents a standard concave arrowhead style with two convex ends and a concave middle, with an average length and width of 115 and 40 nm, respectively. As seen in the TEM images, the synthesized materials are uniform in size and stable in shape. To confirm the detailed structure of the nanoparticles, analyses were performed using high-angle annular dark-field scanning transmission electron microscopy (HAADF-STEM) images (Figure 2D) and high-resolution transmission electron microscopy (HRTEM) images (Figure 2E). As shown by the mapping images in Figure 2E, the Au elements are uniformly distributed. The measured stripe spacing is 0.235 nm, corresponding to the (111) plane of the FCC (face-centered cubic) Au crystal structure, which is used for further simulation.

The crystalline phases of the synthesized AuCNAs were also obtained from XRD measurements. Figure 2F shows that the AuCNAs display four peaks between 30° and 80°, indicating a distinct FCC metal crystal structure. The FCC structure of Au exhibits 2θ values of 39.60°, 45.95°, 64.67°, and 79.81°, corresponding to the (111), (200), (220), and (311) crystal planes, respectively. Distinctly defined strong peaks in the diffractograms demonstrate that the Au nanoparticles exhibit superior crystal quality. Those results suggested that the AuCNAs were successfully prepared.

Based on the results of TEM, the conventional unit cell of the Au crystal, characterized by an FCC structure within the Fm-3m space group, was refined using CASTEP, with the resulting optimized structure presented in Figure 2G. The lattice parameter of the Au unit cell was found to be 0.421 nm, closely matching the data acquired from HAADF-STEM analysis. To produce multilayered two-dimensional Au nanomaterials, bulk Au was cleaved along the (111) crystallographic plane, as depicted in Figure 2H. The interlayer spacing was measured at 0.243 nm, aligning well with the experimental outcomes.

**Figure 3:**
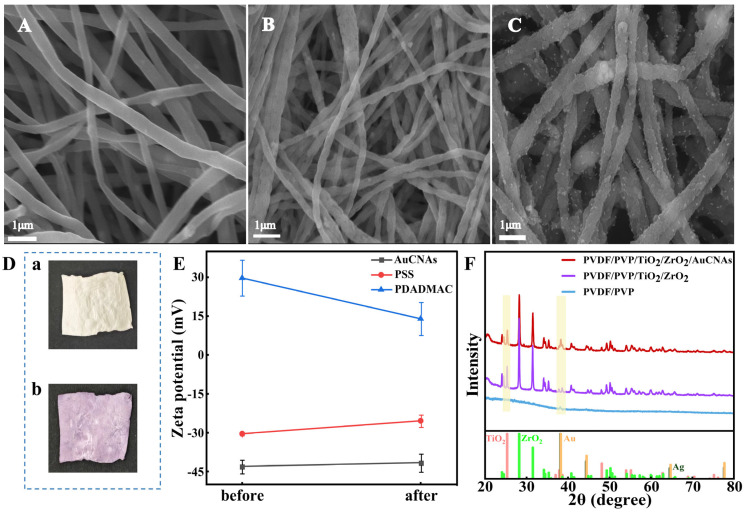
(**A**–**C**) SEM images of nanofiber membranes before and after ultrasound and after assembly; (**D**) nanofiber membrane without AuCNAs loading (a), nanofiber membrane after Au loading (b); (**E**) zeta potential characterization of AuCNAs sol, PDADMAC, and PSS; (**F**) XRD images of three nanofiber membranes with different compositions.

In addition to AuCNAs, the flexible SERS substrates following assembly were characterized. The SEM results reveal that the prepared fibrous membranes possess a continuous and uniform structure. It can be seen in Figure 3A,B that the nanofibers before sonication have a relatively flat and smooth surface before loading AuCNAs. In Figure 3C, a significant number of AuCNAs are attached to the surface of the assembled fibers, confirming that the AuCNAs are successfully loaded. Figure 3D shows the TiO_2_/ZrO_2_ nanofiber membrane, which is white in the actual image and has a relatively smooth surface. After loading AuCNAs, the membrane turns purple, and the surface undulations become more complex.

The sequence of surface charge modifications of electrostatically spun film was successively composed as PDADMAC-PSS-PDADMAC-PSS-PDADMAC. The loading process of AuCNAs on nanofiber membranes was examined; the process variation in assembly by electrostatic adsorption was determined through the potentials of AuCNAs sols, PDADMAC, and PSS/NaCl solutions, respectively. The charge values of the three solutions before and after infiltration are presented in Figure 3E. The surface of the CTAC-encapsulated AuCNAs is positively charged. The zeta potential of the AuCNAs/PSS sol increases from −43.0 mV to −41.57 mV, a change attributed to the interaction between CTAC and PSS. Similarly, the zeta potential of the anionic polyelectrolyte PSS gradually rises from −30.38 mV to −25.39 mV, while the zeta potential of the cationic polyelectrolyte PDADMAC decreases from +29.7 mV to +13.94 mV. Meanwhile, XRD was employed to characterize the nanofiber films with and without TiO_2_, ZrO_2_, and AuCNAs. The results are presented in Figure 3F. The XRD results obtained from the TiO_2_/ZrO_2_ fiber nanofilms exhibit diffraction peaks concentrated at 25.3°, 37.8°, 48.2°, 55.4°, and 62.8°. These peaks are found to correspond to the (101), (004), (200), (105), (211), and (204) crystallographic facets of the tetragonal anatase phase TiO_2_, respectively (JCPDS 76-1938) [57]. The diffraction peaks at 28.2°, 31.5°, 50.2°, 60.0°, and 65.7° are determined to correspond to the (−111), (111), (220), (−302), and (−321) crystal faces of ZrO_2_, respectively (JCPDS 97-008-0048) [58].

### 3.2. Optimization of the Electrostatic Spinning Process

**Figure 4:**
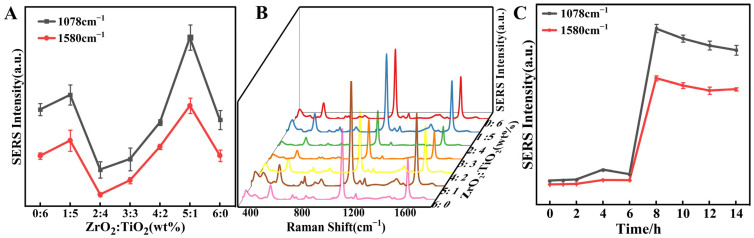
(**A**) Variation curves of the characteristic peak intensity of 4-MBA with polyelectrolyte concentration; (**B**) effect of TiO_2_/ZrO_2_ addition ratio on SERS; (**C**) variation curves of the characteristic peak intensity of 4-MBA with assembly time.

Research has found that the co-doping of TiO_2_ and ZrO_2_ enhances the surface properties of the titanium dioxide phase and increases the specific surface area of the nanofibers [36]. Moreover, when TiO_2_ and ZrO_2_ combine with noble metal nanoparticles (such as Au), they can synergistically enhance the Raman signal through the LSPR effect and the charge-transfer mechanism [55]. Additionally, the loading of nanoparticles on the membrane has an impact on the SERS “hot spots”. Therefore, we explored the effect of the ratio of TiO_2_ and ZrO_2_ on the SERS signal. The SERS signals of 4-MBA represented the optimization results at 10^−6^ M. It is observed that the differences in the SERS signals of 4-MBA obtained with different TiO_2_/ZrO_2_ ratios are particularly conspicuous (Figure 4A). Moreover, Figure 4A,B show that the SERS signals of 4-MBA are highest at the TiO_2_/ZrO_2_ ratio of 1:5. Consequently, the final preparation approach for the electrostatically spun film was the addition of 1% TiO_2_ and 5% ZrO_2_. Meanwhile, an experiment was also carried out to determine the optimum assembly time of the nanoparticles. The SEM of Figure S1 shows that the number of nanoparticles loaded on the electrostatically spun film gradually ascends with the increase in the assembly time, and the nanoparticle loading is almost complete at 8 h. The SERS signal is enhanced accordingly in Figure 4C. After 8 h of assembly, the SERS signal reaches a stable plateau. Meanwhile, the SERS signal at 8 h is also the strongest. In summary, the assembly time of 8 h was chosen as the membrane immersion time for the subsequent experiments.

### 3.3. SERS Performance Verification of Flexible Films

**Figure 5:**
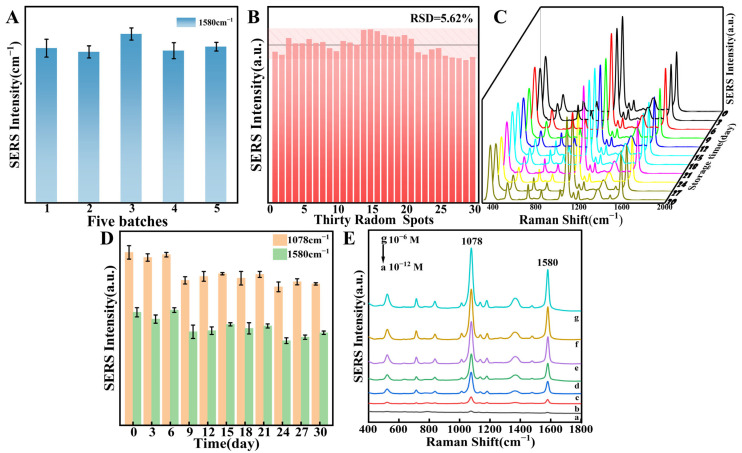
(**A**) The SERS intensity at 1580 cm^−1^ of the characteristic peak of 4-MBA on different batches of membranes; (**B**) SERS intensity maps at 1580 cm^−1^ of the characteristic peak of 4-MBA at 30 sites; (**C**) stability of the flexible substrate SERS signal over a one-month period; (**D**) characteristic peak intensity plot of 4-MBA; (**E**) SERS spectra of 4-MBA at varying concentrations.

In the detection process, the properties of the SERS substrates play a crucial role. Thus, probe molecule 4-MBA was employed to examine the SERS performance of TiO_2_/ZrO_2_/AuCNAs substrates. First, to determine the uniformity of the flexible substrate, SERS signals of 4-MBA at a concentration of 1 μM were collected from 30 different positions on membranes within the same batch and across five different batches. As shown in Figure 5A, the SERS signals of 4-MBA on these five membranes from different batches exhibit minimal variation, with an RSD of 3.65%. This indicates that the material preparation process has excellent inter-batch consistency, and the technical route is feasible for large-scale production, ensuring that substrate materials prepared in different production cycles possess a stable detection performance. Meanwhile, the RSD of the SERS signals measured at 30 points on the surface of the same flexible substrate is 5.62% (Figure 5B), demonstrating that the substrate has good uniformity, with “hot spots” of AuCNAs evenly distributed on the flexible substrate and minimal variation in surface SERS intensity. It is noteworthy that the SERS intensities at the characteristic peaks exhibited less variation over the month (Figure 5C). Figure 5D shows the change in SERS intensity over a one-month period at 1078 and 1580 cm^−1^, with little change in intensity and the RSD below 10%. This observation suggests that the substrate displays excellent robustness. To evaluate the sensitivity of the substrates, SERS measurements were conducted using varying concentrations of 4-MBA (10^−12^~10^−6^ M), as shown in Figure 5E. A decrease in SERS intensity was observed with a corresponding reduction in the concentration of 4-MBA. Furthermore, the EF of the 4-MBA measurement substrate was calculated to be 9.40 × 10^7^, based on Equation (2), which is higher than that of the AuCNAs lysate (EF = 1.1 × 10^6^). Compared with the conventional nanoparticles listed in Table S1, this substrate demonstrates a superior SERS enhancement performance. These results suggest that the TiO_2_/ZrO_2_/AuCNAs substrate, as constructed in this paper, can provide an effective means of subsequent pesticide detection.

### 3.4. SERS Mechanism Exploration of Asomate on Flexible Film

**Figure 6:**
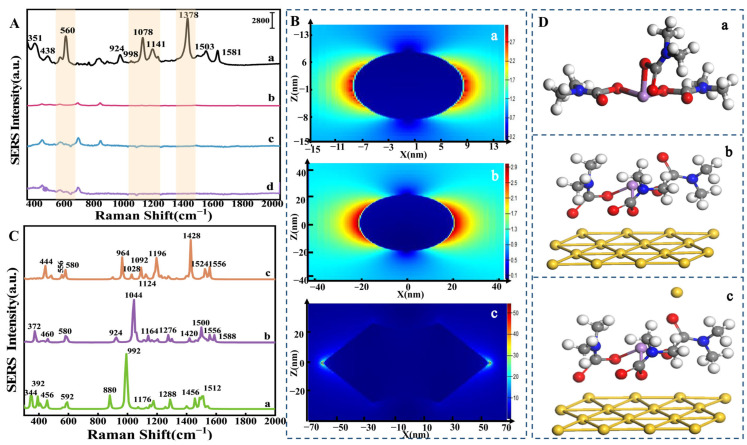
(**A**) Detection of asomate solution by flexible substrate with or without AuCNAs: (a) TiO_2_/ZrO_2_, (b) TiO_2_/ZrO_2_ + asomate, (c) TiO_2_/ZrO_2_/AuCNAs, (d) TiO_2_/ZrO_2_/AuCNAs+ asomate; (**B**) simulated distributions of electromagnetic field intensity: (a) TiO_2_, (b) ZrO_2_, and (c) AuCNAs; (**C**) comparison of simulated Raman signals of (a) asomate, (b) asomate adsorbing Au surface, and (c) asomate adsorbing Au atom and Au surface; (**D**) molecular simulation results corresponding adsorption states for (a) asomate, (b) asomate adsorbing Au surface, and (c) asomate adsorbing Au atom and Au surface.

To gain a deeper understanding of the mechanisms underlying the SERS signals of flexible substrates, this paper focuses on the role of AuCNA-stabilized metal nanoparticles and semiconductor-natured substance nanoparticles, including TiO_2_ and ZrO_2_. Based on this, flexible substrates with TiO_2_/ZrO_2_ and TiO_2_/ZrO_2_/AuCNAs were prepared, and the corresponding SERS signals with and without asomate (1 µM) are presented in Figure 6A. Figure 6A(a) depicts the SERS peaks generated by the interaction of 1 µM asomate with TiO_2_/ZrO_2_/AuCNA substrates. It can be observed that asomate exhibits prominent peaks at 560, 1078, and 1378 cm^−1^. In contrast to the flexible substrate with TiO_2_/ZrO_2_/AuCNA, the flexible substrate with TiO_2_/ZrO_2_ exhibits low SERS activity, exhibiting only weak peaks in its substrate. This finding indicated that the AuCNAs may play a pivotal role in the SERS signals of the flexible substrate. This phenomenon is believed to arise from the LSPR effect.

Due to the LSPR effect reflected as the strength of the electric field [59,60,61], the FDTD method [62] was employed to simulate the electric field distributions around the nanoparticles, including TiO_2_, ZrO_2_, and AuCNAs. The sizes and shapes of the TiO_2_, ZrO_2_, and AuCNAs nanoparticles were obtained through the results of TEM (Figure S2). Based on the shapes and sizes of the nanoparticles, the FDTD simulation was executed. The results of the FDTD are presented in Figure 6B, in which the maximum electric field strengths generated by the three nanoparticle models in the XZ direction were 3.12 V/nM, 2.96 V/nM, and 54.2 V/nM. The AuCNAs exhibit the strongest electric field intensity, confirming our hypothesis.

After analyzing the LSPR effect, the relationship between the SERS signal and the structure of asomate was investigated. In this study, a three-layer Au (111) surface was fabricated, and the CASTEP module, grounded in density functional theory, was employed to geometrically optimize the configuration of asomate adsorbed onto the Au (111) surface. Subsequently, the adsorption process of asomate molecules onto the Au surface was simulated. The lowest adsorption configuration, which became the cornerstone of our subsequent studies, is captured in Figures S3 and S4. In this configuration, the three S atoms neighboring As are precisely aligned above the Au atoms, with an interlayer distance of 0.245 nm. Substantial changes in bond lengths and angles were detected after adsorption, in contrast to the asomate molecule before adsorption, indicating a strong interaction between the asomate molecule and the Au (111) surface. Adsorption energy, a key parameter for material stability, was calculated to be −11.9 eV for this configuration, suggesting the stability of the structure. After obtaining the lowest energy configuration, simulations were conducted using Gaussian 16 software to model the adsorption of asomate molecules on the Au (111) surface, enabling the acquisition of the Raman spectrum. To reduce computational load, only the surface atoms of the Au nanoparticle close to the asomate molecule were included in the simulations, as these adjacent Au atoms can strongly interact with the asomate molecule.

After obtaining the structure of asomate absorbed to the Au surface, the Raman spectra were simulated. During simulation, three structures were constructed for comparison, including a single asomate molecule (Aso), an asomate molecule with an Au surface (Aso+Ausu), and an asomate molecule with an Au surface and a disassociated Au atom (Aso+Ausu+Agdisso) (Figure 6D). Meanwhile, the corresponding simulated Raman spectra can be shown in plot C of Figure 6. In line a of plot C, the peaks of the Raman signal for the asomate molecule are largely different from the practical SERS signal, as in line a in plot A of Figure 6, there are no large peaks between 1288 cm^−1^ and 1456 cm^−1^, where practical SERS shows a strong peak at 1378 cm^−1^. Meanwhile, the simulated Raman signal of asomate exhibits nearly no peak at regions of 992~1176 cm^−1^, while the practical SERS has some strong peaks at this region, such as the one at 1308 cm^−1^. We guess the difference may be related to the interaction between the asomate molecule and the Au surface. Thus, the Raman signal of Aso+Ausu is simulated and illustrated as line b in plot C. Compared to line a, the Raman signal for Aso+Ausu in line b closely resembles the practical SERS signal. Notably, a strong peak is observed at 1044 cm^−1^, which aligns closely with the peak at 1078 cm^−1^. Additionally, the peaks at 580 cm^−1^ and 924 cm^−1^ in line b are close to those observed at 560 cm^−1^ and 824 cm^−1^ in the practical SERS spectrum.

Similar to our previous research [63], we guessed that the disassociated Au atom may also influence the SERS signals. Thus, the Raman signal of Aso+Ausu+Audisso was simulated and is shown as line c in plot C. Compared with line b in plot C, the Raman signal of line c is closer to that of practical SERS. The prominent Raman peaks at 444, 556, and 1092 cm^−1^ closely correspond to the practical SERS peaks observed at 438, 560, and 1078 cm^−1^, respectively. Additionally, in line c of plot C, a strong peak is detected at 1428 cm^−1^, which falls within the range of 1388~1444 cm^−1^, alongside a shoulder peak at 1390 cm^−1^. Those simulated peaks may be related to the strong peak of practical SERS in plot A at 1378 cm^−1^. The findings revealed that the Raman simulation peaks obtained from the interaction among asomate molecule, disassociated Au atoms, and Au facet are more closely aligned with the practical SERS peaks for detecting asomate (Table S2).

### 3.5. Detection of Asomate on Apple Epidermis

**Figure 7:**
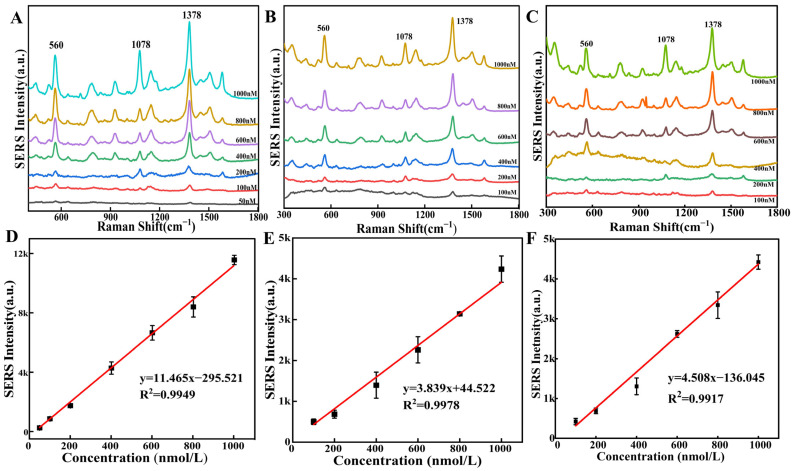
The SERS spectra of asomate at various concentrations measured on a flexible substrate in the standard solutions (**A**), on the skin of Red Delicious apples (**B**), and on the skin of Cream Fuji apples (**C**); the linear relationships between the SERS intensities at 1378 cm^−1^ and the asomate concentrations in the standard solutions (**D**), on the skin of Red Delicious apples (**E**), and on the skin of Cream Fuji apples (**F**).

The SERS intensities of different concentrations (50~1000 nM) of asomate standard solutions on flexible substrates of TiO_2_/ZrO_2_/AuCNAs were initially examined. As shown in Figure 7A and Figure S5, the characteristic peaks at 560, 1078, and 1378 cm^−1^ are observable at a concentration of 50 nM. The characteristic peak at 1378 cm^−1^ with the largest intensity underwent a linear fit of SERS intensity versus concentration, as depicted in Figure 7D, with the linear equation as *y* = 11.464*x* − 295.521, *R*^2^ = 0.994. These results suggested the feasibility of using the flexible substrate to detect the asomate solution.

To verify the practical acquisition and detection capabilities of the flexible substrates of TiO_2_/ZrO_2_/AuCNAs and to determine their suitability for application in actual pesticide residue analysis, asomate residues were detected on apple peel using the flexible substrates. In situ detection was performed by applying drops of asomate standards of varying concentrations (100~1000 nM) to the apple peel. As illustrated in Figure 7B, the asomate collected from the surface of Red Delicious apples has distinct characteristic peaks, and the intensity of these peaks exhibited a gradual decline in conjunction with the reduction in concentration. The distinctive peak at 1378 cm^−1^ remains discernible when the concentration of asomate on the apple surface was as low as 100 nM. The result demonstrated that the flexible substrate could be used to detect residual asomate on the surface of apples at extremely low levels. The SERS intensities and concentrations at the characteristic peak 1378 cm^−1^ were subjected to linear fitting (Figure 7E), resulting in the following equation: *y* = 3.839*x* + 44.522, *R*^2^ = 0.9978. The LOD measured by this method was 9.62 nM. Similarly, the residues of asomate on the skin of Cream Fuji apples were also detected. The changes in the obtained SERS signals are shown in Figure 7C. Figure 7F presents the linear equation between the SERS intensities at the characteristic peak of 1378 cm^−1^ and the concentrations of the collected asomate. The equation is *y* = 4.508*x* − 44.522, with an *R*^2^ = 0.9917. The measured LOD is 8.19 nmol/L. These results indicate that the prepared TiO_2_/ZrO_2_/AuCNAs flexible substrate has an excellent detection performance. Moreover, the low LOD confirms the feasibility of detecting actual fungicide residues, demonstrating that the flexible substrate exhibited an excellent detection performance. Furthermore, the lower detection limit substantiated the feasibility of practical fungicide residue detection.

Table S3 summarizes the preceding research findings on the test of dithiocarbamate fungicides, as referred to in the literature [54,64,65,66,67,68,69]. In comparison to alternative SERS methodologies, the detection threshold in this investigation is markedly diminished and is even more sensitive than the detection limits of certain established technologies. Furthermore, in Table 1, the recovery rates of Red Delicious apples range from 95.94 to 102.84%, while those of Cream Fuji apples fall between 99.22 and 111.21%. Both recovery rates meet the standards for trace analysis, indicating that SERS has high accuracy for detecting asomate in actual samples.

**Figure 8:**
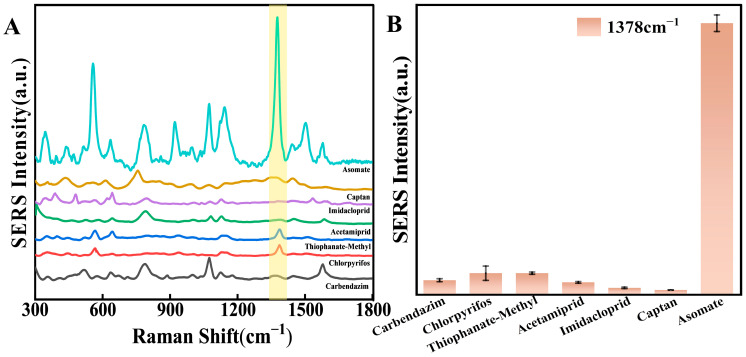
Interference data on flexible SERS substrates (**A**) Raman curves, (**B**) SERS intensity maps of the characteristic peaks at 1378 cm^−1^.

To further assess the feasibility and utility of flexible substrates of TiO_2_/ZrO_2_/AuCNAs, their selectivity was investigated against other commonly used apple fungicides. For this purpose, carbendazim, chlorpyrifos, thiophanate-methyl, acetamiprid, imidacloprid, and captan were analyzed using the same assay conditions as the above fungicides. As illustrated in Figure 8A,B, high-intensity SERS signals are observed at 1378 cm^−1^ at a concentration of 400 nM of asomate. In contrast, only low SERS signals were recorded for the other fungicides, even at a concentration of 4 μM. It suggested that the SERS substrate could selectively detect asomate at 1378 cm^−1^.

## 4. Conclusions

In summary, the flexible SERS substrates for asomate detection were achieved by utilizing electrostatic spinning technology. A sensitive and flexible TiO_2_/ZrO_2_/AuCNAs SERS substrate was fabricated by loading AuCNAs onto the modified TiO_2_/ZrO_2_ electrospinning film via electrostatic adsorption. The substrate was then applied to detect asomate in the apple epidermis. The results demonstrated that the flexible SERS substrate exhibited a remarkable Raman enhancement effect, outstanding homogeneity, and exceptional stability. Based on asomate SERS signals in actual Red Delicious and Cream Fuji apples, good linear correlations were observed in the concentration range of 100~1000 nmol/L, with R^2^ values of 0.9978 and 0.9917, respectively. The LODs were 9.62 and 8.19 nmol/L, respectively, highlighting the significant potential of the flexible substrate for the in situ detection of pesticide residues. Moreover, the mechanism of the asomate SERS signal was investigated by the FDTD and DFT. The FDTD exhibited that the LSPR effects related to the strength of the SERS signal are mainly generated from the ‘hot spots’ in AuCNAs. The DFT simulation revealed that SERS peaks are generated by asomate molecules lying on the Au surface that absorb Au atoms. Nevertheless, practical challenges persist regarding the substrate’s stability under harsh environmental conditions. Long-term storage reliability and performance maintenance in extreme environments (e.g., high temperature and strong acids/bases/oxidants) require further investigation. These limitations may affect real-world deployment in variable agricultural settings. Although these flexible SERS substrates with uniformity and easy preparation have been used to detect asomate residues, future research endeavors should concentrate on reusable substrates, intending to reduce manufacturing costs, optimize manufacturing methodologies, and facilitate commercialization.

## Figures and Tables

**Table 1:** Standard recovery test of asomate residue on the apple surface.

Types	Spiked (nM)	Detected (nM)	Recovery (%)	RSD (%)
Red Delicious Apple	300	307.75	102.58	4.89
	500	479.71	95.94	3.16
	700	719.87	102.84	7.76
Cream Fuji Apple	300	333.62	111.21	6.65
	500	519.32	103.86	8.38
	900	892.94	99.22	2.27

## Data Availability

The original contributions presented in the study are included in the article/Supplementary Materials, further inquiries can be directed to the corresponding authors.

## Supplementary Materials

The following supporting information can be downloaded at: https://www.mdpi.com/article/10.3390/foods14122062/s1: Figure S1. SEM images of nanofiber membranes with different AuCNAs self-assembly times: A–H are 1, 2, 4, 6, 8, 10, 12, and 14 h, respectively; Figure S2. TEM images of TiO_2_ (A), ZrO_2_ (B), and AuCNAs (C). Figure S3. The model of gold surface adsorbing asomate molecules was constructed based on the Au (111) facet. Figure S4. The lowest energy states of the gold surface adsorbing asomate molecules exhibit planar orientation from top view (A), front view (B), and side view (C). Figure S5. The linear correlation analysis between asomate concentrations and intensities of SERS peaks at (A) 560, (B) 924, (C) 1078, (D) 1141, (E) 1378, and (F) 1503 cm^−1^. Table S1. Comparison of EF with different SERS substrates. Table S2. Assignments of main theoretical and experimental SERS peaks for asomate. Table S3. Summarization of the results of previous research on the determination of dithiocarbamates.
