# Supplementary material for: Flexible TiO2/ZrO2/AuCNAs Surface-Enhanced Raman Scattering Substrates for the Detection of Asomate in Apple Peel
Source: Foods. 2025 Jun 11;14(12):2062. doi: 10.3390/foods14122062 (PMC12191852; doi:10.3390/foods14122062)
Supplement: Supplementary file 1 [file foods-14-02062-s001.zip › supplement20250602.pdf]

Flexible TiO_2/ZrO_2/AuCNAs surface-enhanced Raman scattering substrates for the detection of asomate in apple peel

Lina Zhao [1], Zhengdong Sun [2] Ye Shen[a], Zhiyang Chen[1], Yang Zhang[1], Jiyong Shi[1] Haroon, Elrasheid[1], Xuechao Xu[3], Kaiyi Zhenga[1]*, Xiaobo Zou[1]* and Meng Zhang[2]*

1 School of Food and Biological Engineering, Jiangsu University, Zhenjiang, Jiangsu, 212013, China; kai-yizheng@ujs.edu.cn (K. Zheng); zou_xiaobo@ujs.edu.cn (X. Zou)

2 Department of Physics, East China University of Science and Technology, Shanghai, 200237, China; mzhang@ecust.edu.cn (M. Zhang)

3 School of Food Science and Engineering, Yangzhou University, Yangzhou, 225127, Jiangsu, China; xuechao-xu@yzu.edu.cn (X. Xu)

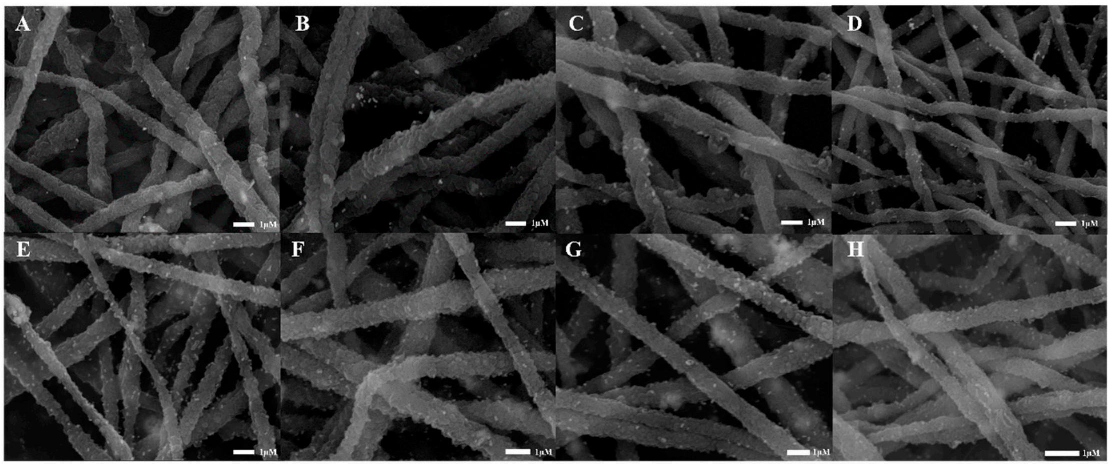

Figure S1. SEM images of nanofiber membranes with different AuCNAs self-assembly times: A-H are 1, 2, 4, 6, 8, 10, 12, and 14 hours, respectively.

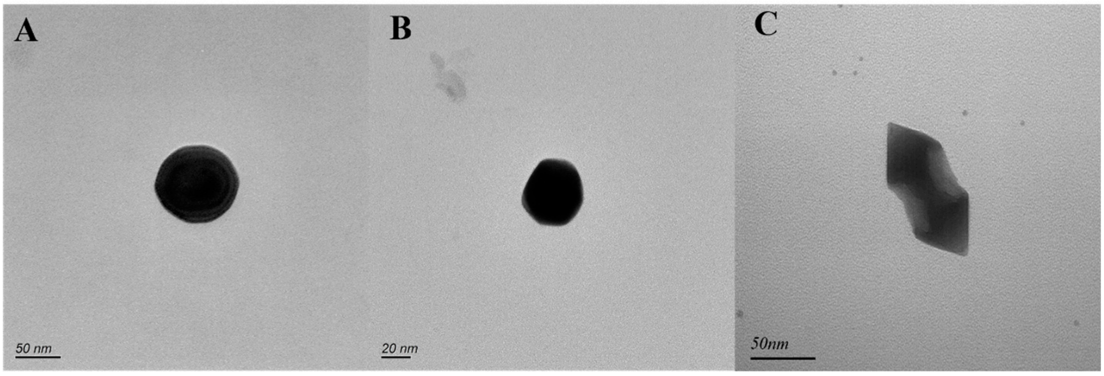

Figure S2 TEM images of TiO_2 (A), ZrO_2 (B) and AuCNAs (C).

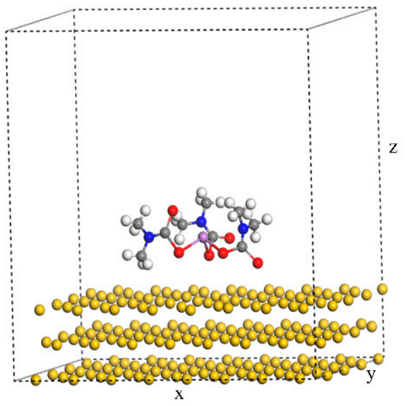

Figure S3. The model of gold surface adsorbing asomate molecule was constructed based on the Au (111) facet.

Mulliken atomic charges: Asomate:0.0280e, Au:-0.0320e

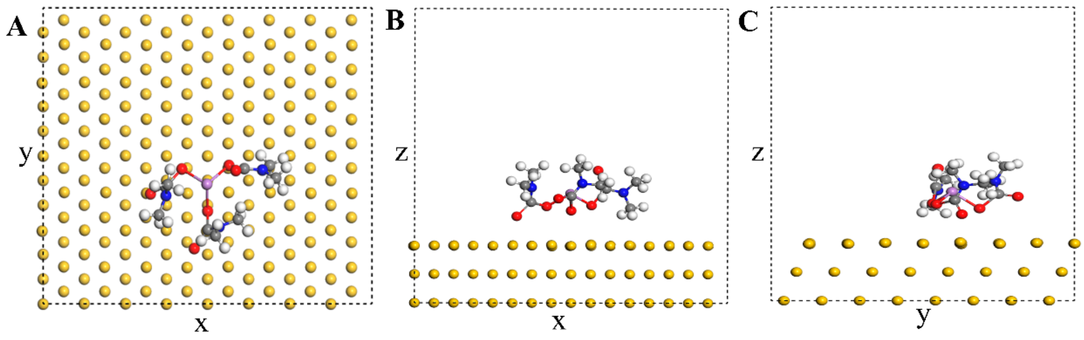

Figure S4. The lowest energy states of the gold surface adsorbing asomate molecule exhibit planar orientation from top view (A), front view (B) and side view (C).

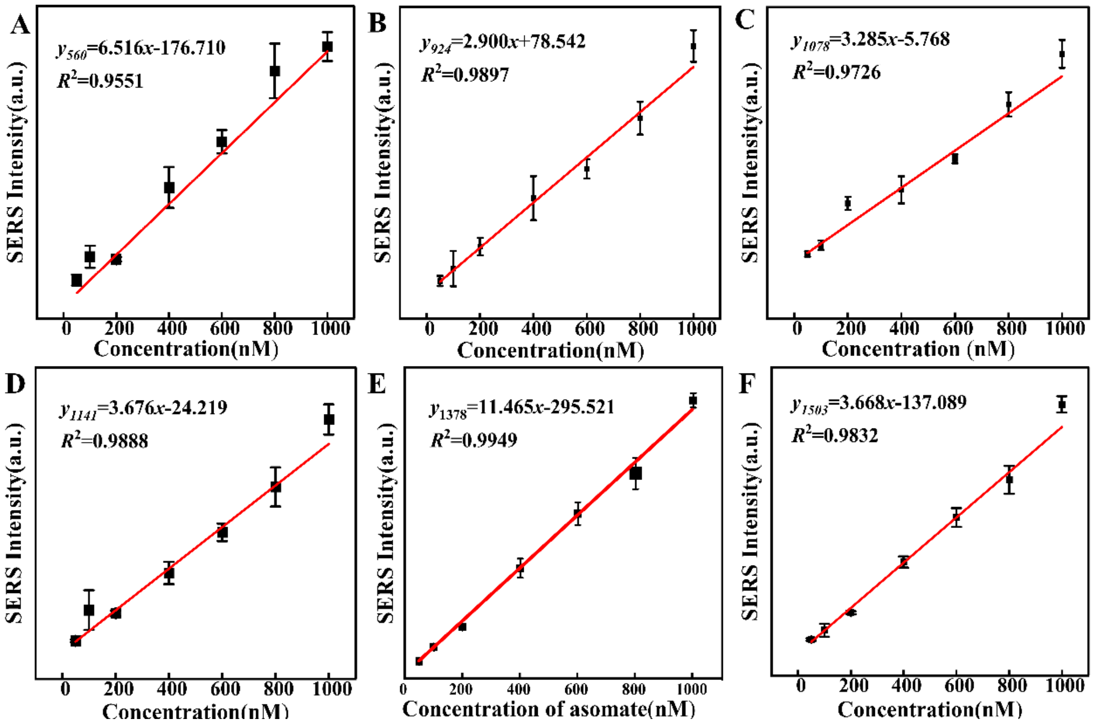

Figure S5. The linear correlation analysis between asomate concentrations and intensities of SERS peaks at (A) 560, (B) 924, (C) 1078, (D) 1141, (E) 1378 and (F) 1503 cm^{-1}.

Table S1 Comparison of EF with different SERS substrates

Nanomaterials	AuCNAs	AgNRs	Ag nanoparticles	AuNSs
EF	9.40×10^7	6.56×10^5	4.44×10^5	4.29×10^5

Table S2. Assignments of main theoretical and experimental SERS peaks for asomate.

Theoretical (cm^{-1})	SERS (cm^{-1})	Assignment
444	438	H_3C-N-CH_3 swing
556	560	C-N swing vibration
964	924	CH_3-N and C-S telescopic vibration
1028	998	C-N bending
1092	1078	C-H bending
1124	1141	C-H in-plane rocking
1428 (wide and strong peak)	1378 (wide and strong peak)	-CH_3 deformation and C-N telescopic vibration
1524	1503	C-H swing
1556	1581	C-H swing

Table S3. Summarization for the results of previous researches on the determination of dithiocarbamates.

References	Method	Pesticide	Samples	LOD（nM）
[1]	RP-HPLC	Asomate	Apple	677.96
[2]	HPLC	Asomate	Soil	229
[3]	SERS	Thiram	Apple	91.50
[4]	SERS	Thiram	Milk	70.88
[5]	SERS	Mancozeb	Tomato	200
[6]	SERS	Thiram	Apple	74.86
[7]	SERS	Ferbam	Water	38
This work	SERS	Asomate	Apple	9.62

References

1. Niu, P.F.; Shen, Y.; Li, S.; Guo, Y.R.. Determination of asomate residue in spples by RP-HPLC. *Jiangsu Journal of Agricultural Sciences* **2018**, *34*, 706-710.
2. Huang, P.X.; Liu, X.W.; Wang, L.; Peng, Y.; Luo M.. Determination of asomate in apples and soils by high herformance liquid chromatography. *Chinese Journal of Analysis Laboratory* **2016**, *35*, 86-89, doi:https://doi.org/10.13595/j.cnki.issn1000-0720.2016.0020.
3. Guo, Z.M.; Zheng, Y.X.; Yin, L.M.; Xue, S.S.; Ma, L.X.; Zhou, R.Y.; El-Seedi, H.R.; Zhang, Y.; Yosri, N.; Jayan, H.; et al. Flexible Au@AgNRs/MAA/PDMS-based SERS sensor coupled with intelligent algorithms for in-situ detection of thiram on apple. *Sens. Actuator B-Chem.* **2024**, *404*, 10, doi:https://doi.org/10.1016/j.snb.2024.135303.
4. Wang, J.J.; Luo, Z.S.; Lin, X.Y. An ultrafast electrochemical synthesis of Au@Ag core-shell nanoflowers as a SERS substrate for thiram detection in milk and juice. *Food Chemistry* **2023**, *402*, 8, doi:https://doi.org/10.1016/j.foodchem.2022.134433.
5. Tsen, C.M.; Yu, C.W.; Chen, S.Y.; Lin, C.L.; Chuang, C.Y. Application of surface-enhanced Raman scattering in rapid detection of dithiocarbamate pesticide residues in foods. *Appl. Surf. Sci.* **2021**, *558*, 11, doi:https://doi.org/10.1016/j.apsusc.2021.149740.
6. Pu, H.B.; Huang, Z.; Xu, F.; Sun, D.W. Two-Dimensional Self-Assembled Au-Ag Core-Shell Nanorods Nanoarray for Sensitive Detection of Thiram in Apple Using Surface-Enhanced Raman Spectroscopy. *Food Chemistry* **2021**, 343.
7. Zhu, C.H.; Wang, X.J.; Shi, X.F.; Feng, Y.; Guowen, M.; Qizhong, X.; Yan, K.; Hua, W.; Yilin, L.; Nianqiang, W. Detection of Dithiocarbamate Pesticides with a Spongelike Surface-Enhanced Raman Scattering Substrate Made of Reduced Graphene Oxide-Wrapped Silver Nanocubes. *ACS applied materials & interfaces* **2017**, *9*, 39618-39625, doi:https://doi.org/10.1021/acsami.7b13479.
