# Supplementary figures and images for: Flexible TiO2/ZrO2/AuCNAs Surface-Enhanced Raman Scattering Substrates for the Detection of Asomate in Apple Peel
Source: Foods. 2025 Jun 11;14(12):2062. doi: 10.3390/foods14122062 (PMC12191852; doi:10.3390/foods14122062)

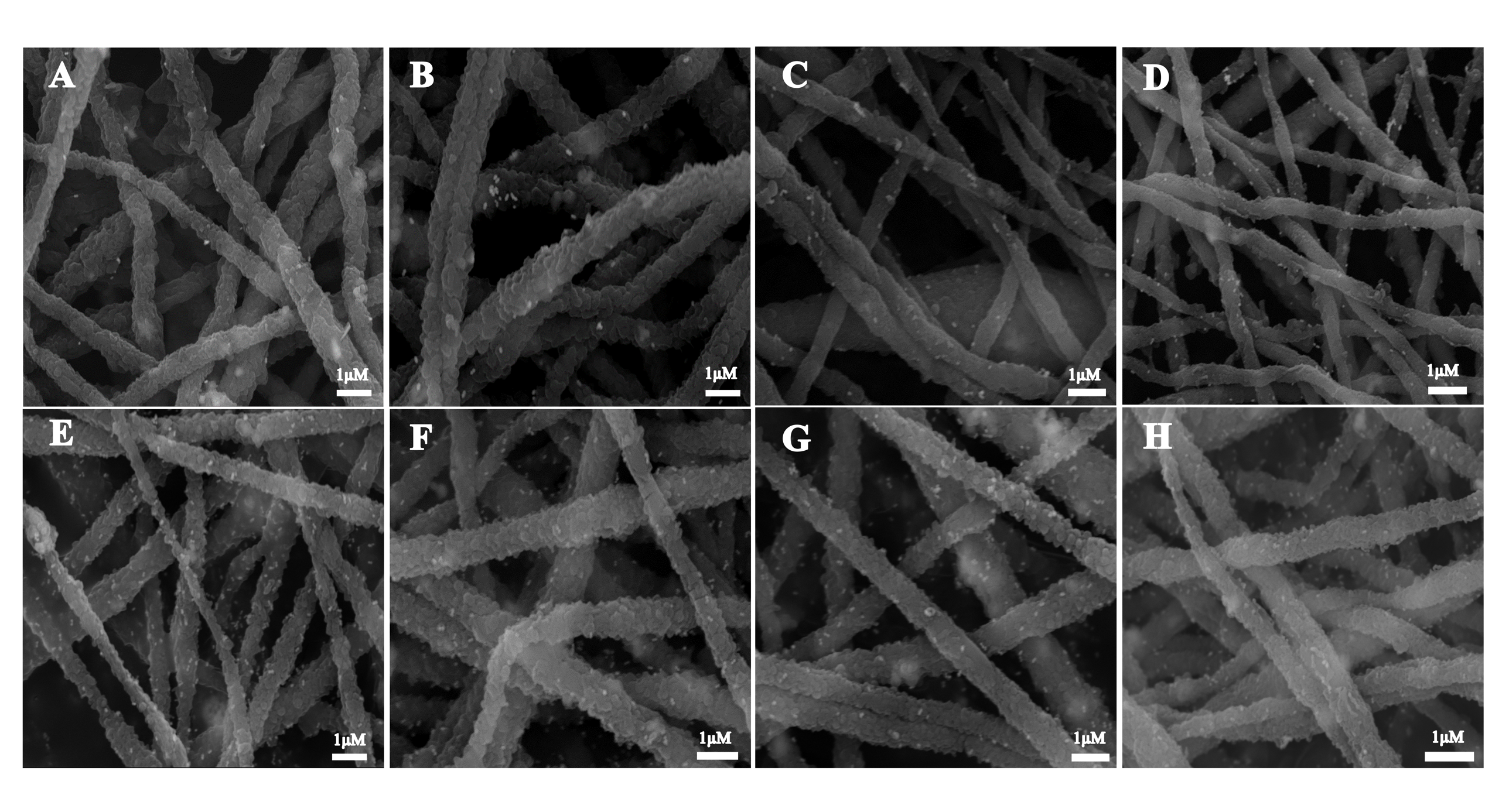

Supplement: Supplementary file 1 [file foods-14-02062-s001.zip › Fig. S1.tif]

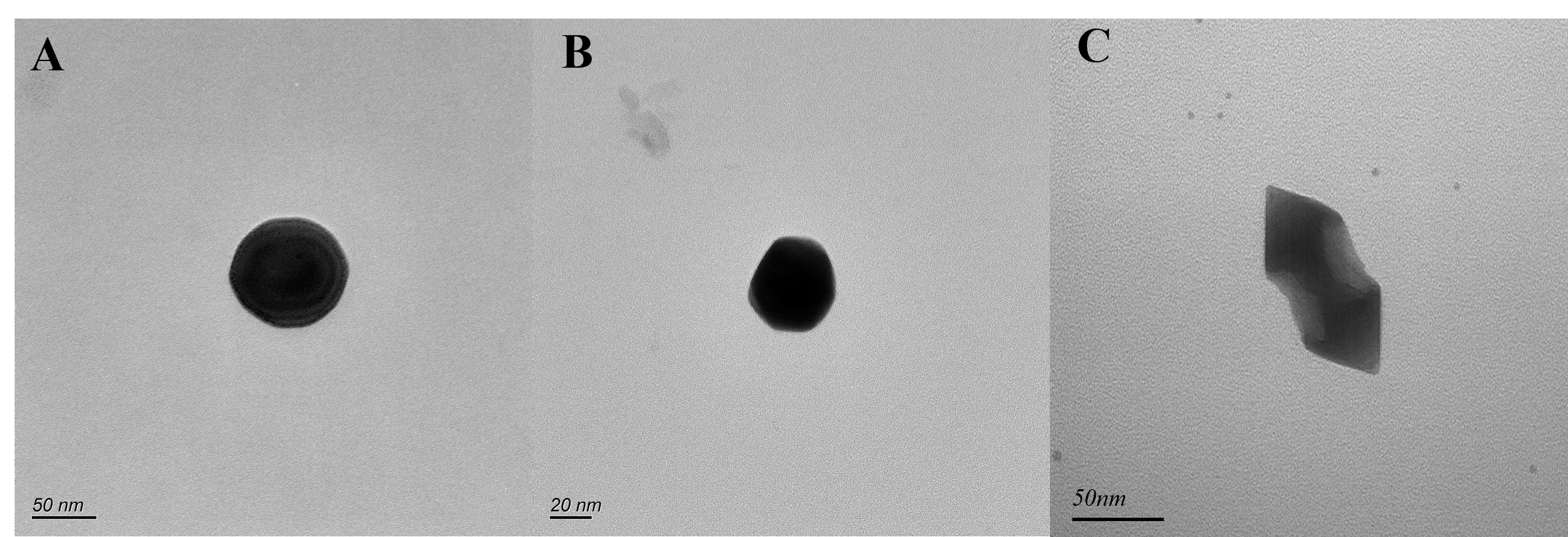

Supplement: Supplementary file 1 [file foods-14-02062-s001.zip › Fig. S2.tif]

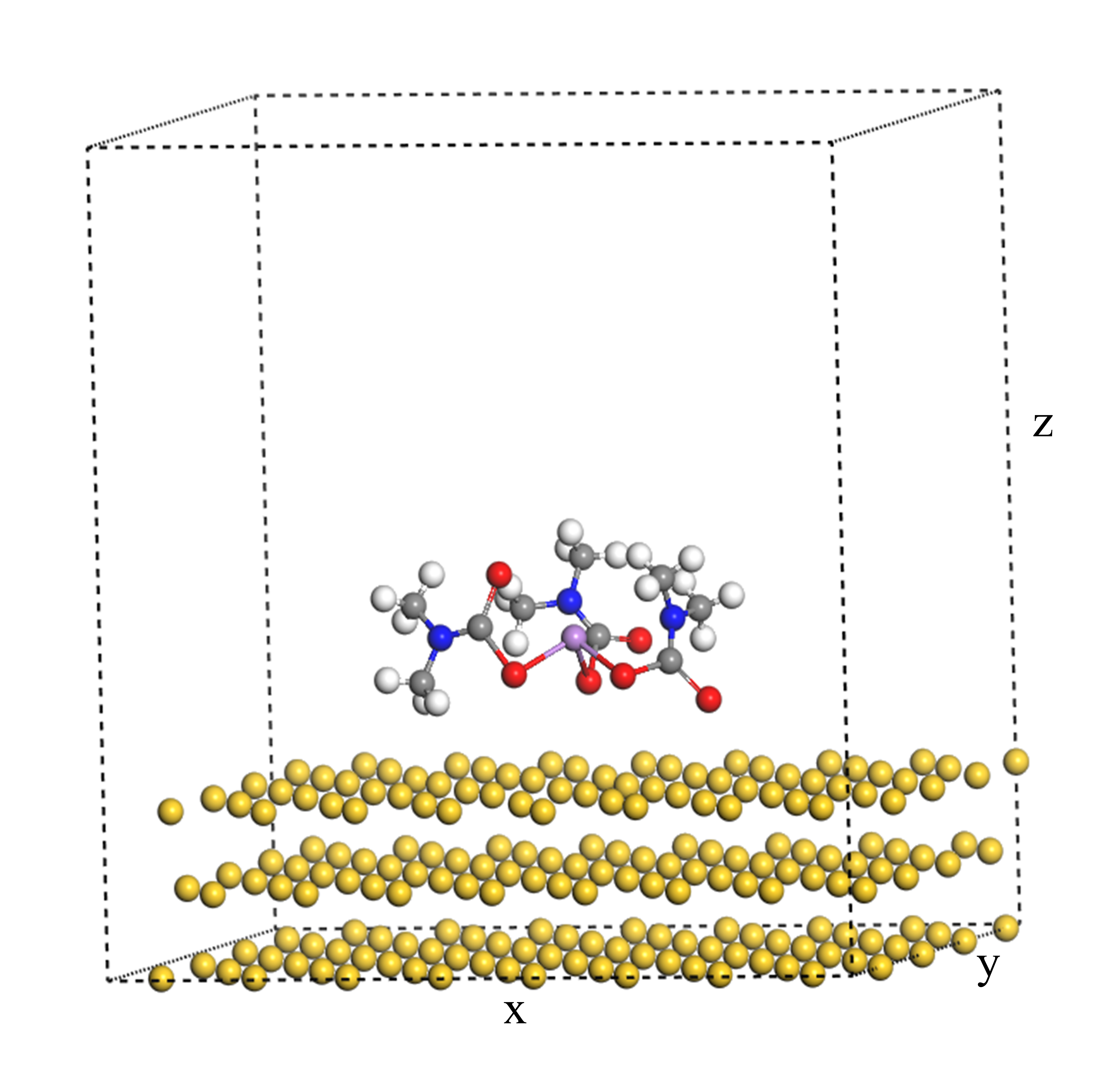

Supplement: Supplementary file 1 [file foods-14-02062-s001.zip › Fig. S3.tif]

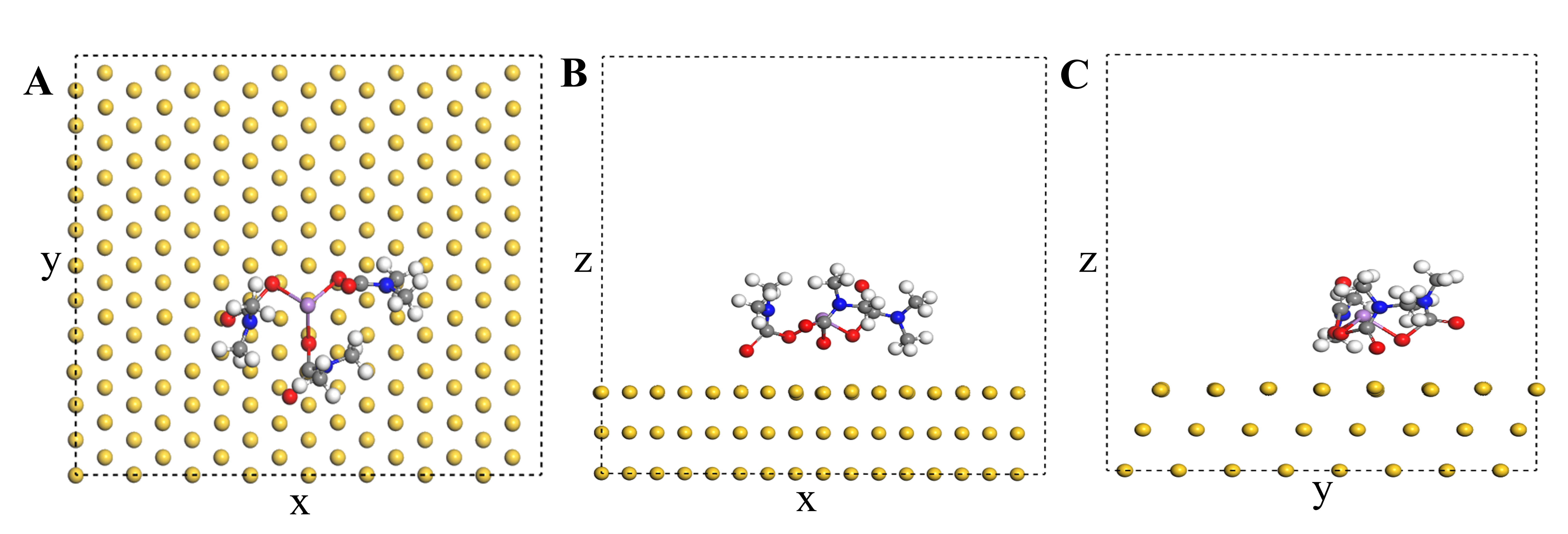

Supplement: Supplementary file 1 [file foods-14-02062-s001.zip › Fig. S4.tif]

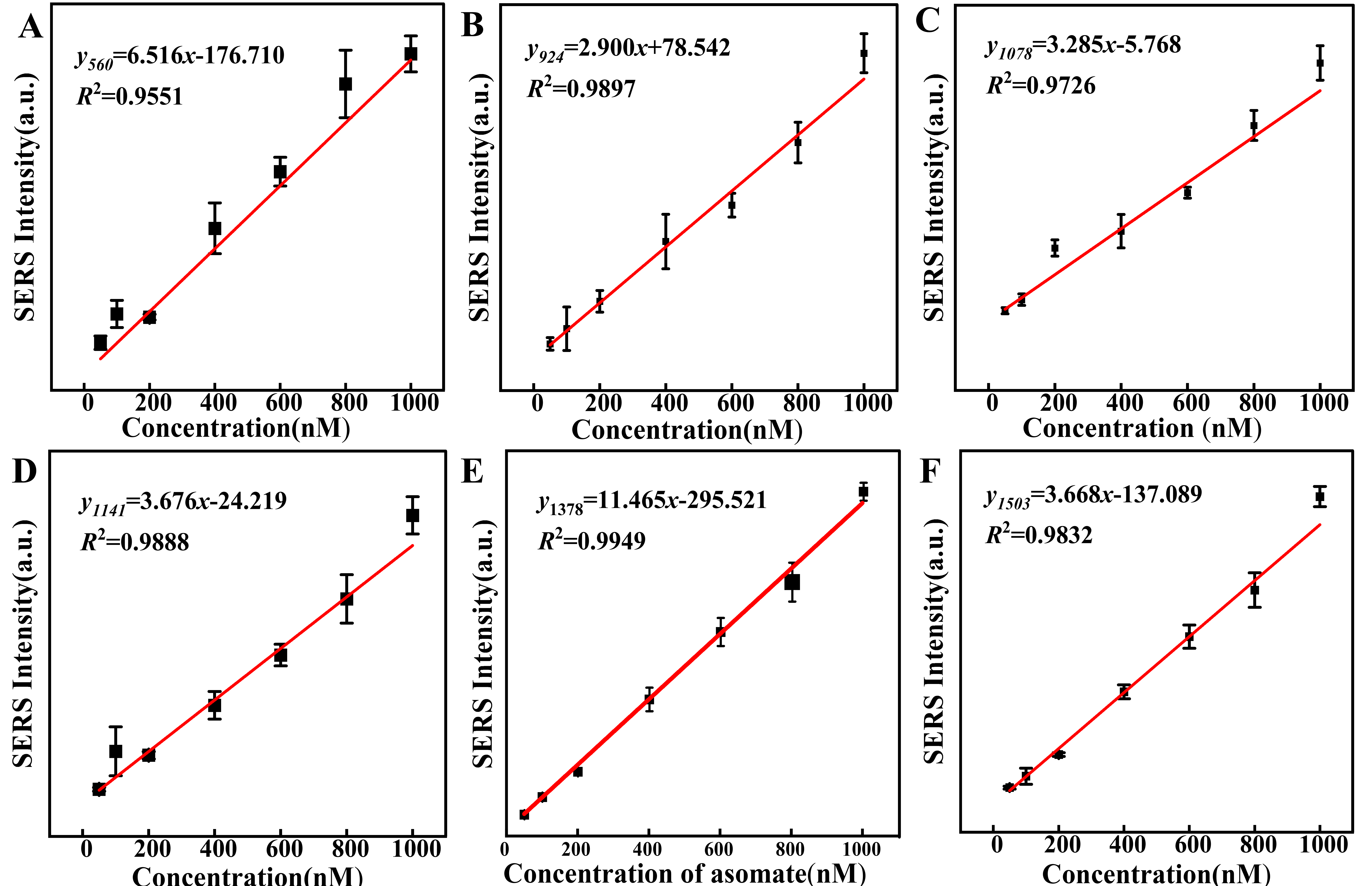

Supplement: Supplementary file 1 [file foods-14-02062-s001.zip › Fig. S5.tif]
